# Identification of immune-related molecular clusters and diagnostic markers in chronic kidney disease based on cluster analysis

**DOI:** 10.3389/fgene.2023.1111976

**Published:** 2023-02-06

**Authors:** Peng Yan, Ben Ke, Jianling Song, Xiangdong Fang

**Affiliations:** Department of Nephrology, The Second Affiliated Hospital of Nanchang University, Nanchang, China

**Keywords:** chronic kidney disease, immune, IRGs, molecular clusters, machine learning, biomarker

## Abstract

**Background**: Chronic kidney disease (CKD) is a heterogeneous disease with multiple etiologies, risk factors, clinical manifestations, and prognosis. The aim of this study was to identify different immune-related molecular clusters in CKD, their functional immunological properties, and to screen for promising diagnostic markers.

**Methods**: Datasets of 440 CKD patients were obtained from the comprehensive gene expression database. The core immune-related genes (IRGs) were identified by weighted gene co-expression network analysis. We used unsupervised clustering to divide CKD samples into two immune-related subclusters. Then, functional enrichment analysis was performed for differentially expressed genes (DEGs) between clusters. Three machine learning methods (LASSO, RF, and SVM-RFE) and Venn diagrams were applied to filter out 5 significant IRGs with distinguished subtypes. A nomogram diagnostic model was developed, and the prediction effect was verified using calibration curve, decision curve analysis. CIBERSORT was applied to assess the variation in immune cell infiltration among clusters. The expression levels, immune characteristics and immune cell correlation of core diagnostic markers were investigated. Finally, the Nephroseq V5 was used to assess the correlation among core diagnostic markers and renal function.

**Results**: The 15 core IRGs screened were differentially expressed in normal and CKD samples. CKD was classified into two immune-related molecular clusters. Cluster 2 is significantly enriched in biological functions such as leukocyte adhesion and regulation as well as immune activation, and has a severe immune prognosis compared to cluster 1. A nomogram diagnostic model with reliable prediction of immune-related clusters was developed based on five signature genes. The core diagnostic markers LYZ, CTSS, and ISG20 were identified as playing an important role in the immune microenvironment and were shown to correlate meaningfully with immune cell infiltration and renal function.

**Conclusion**: Our study identifies two subtypes of CKD with distinct immune gene expression patterns and provides promising predictive models. Along with the exploration of the role of three promising diagnostic markers in the immune microenvironment of CKD, it is anticipated to provide novel breakthroughs in potential targets for disease treatment.

## Introduction

Chronic kidney disease (CKD) is a highly heterogeneous disease with multiple etiologies, risk factors, and outcomes, that is commonly defined as the persistence of structural or functional abnormalities of the kidney for greater than 3 months that severely affect health. CKD has been recognized as one of the world’s growing public health problems. According to the Global Burden of Kidney Disease (2017), there are 697.5 million CKD cases worldwide, almost a third of which are in China and India, and the total number of individuals receiving kidney replacement therapy is over 2.5 million and is expected to double by 2030, making it a major health and economic challenge across regions (Bikbov et al., 2020). The prevalence of CKD is still increasing, and clinical outcomes are unsatisfactory. Very often, the burden of CKD is not restricted to the impact on the need for renal replacement therapy after progression to end-stage renal disease (ESRD). In fact, patients with CKD tend to have a significantly increased risk of cardiovascular disease and are at higher risk of hospitalization and death (Webster et al., 2017). And since there are no symptoms in the early stages, as many as nine out of ten adults with CKD are unaware of their kidney damage, and CKD acts as a “silent killer”. Despite the high burden and impact caused by the disease, available therapy options to stop or slow the progression of CKD continue to be limited. This may be attributed to the fact that the underlying mechanisms of CKD pathogenesis and progression are not fully understood. Therefore, in-depth study of CKD-related pathologies and understanding of its underlying molecular mechanisms are important for early diagnosis of the disease and providing new therapeutic targets.

Influencing the rate of progression in CKD patients is usually associated with genes polymorphisms of inflammatory response, fibrosis, metabolism, CKD progression, and renin-angiotensin-aldosterone system (RAAS) (Yan et al., 2021). Notably, there is mounting evidence that renal immune dysregulation plays a critical role in the physiological dysfunction and development associated with CKD (Espi et al., 2020; Tang et al., 2021). Regardless of the underlying etiology, the inflammatory response is continuously activated in CKD, characterized by the recruitment of immune cell throughout the kidney, along with local overproduction of growth factors (e.g., tumor necrosis factor (TNF)-α, interleukins, interferon (IFN)-γ, chemokines) and pro-fibrotic cytokines (Suárez-Fueyo et al., 2017; Cantero-Navarro et al., 2021). Under this regulation, different cellular and molecular processes are activated, leading to persistent kidney injury and disease progression in CKD. Therefore, exploring the correlation between immune-related genes and CKD progression may help to elucidate the pathogenesis of CKD patients at the molecular level. However, little is known about the precise regulatory genetic and molecular mechanisms of immune involvement in the pathogenesis and progression of CKD.

Thanks to the rise and advancement of bioinformatics analysis and high-throughput sequencing, new approaches will contribute to a better understand the biology of kidney disease through the use of genetic, epigenetic modification and transcriptomic studies, providing new clues and opening up new unknown avenues for the study of disease mechanisms (Pareek et al., 2011; O’seaghdha and Fox, 2011). Several bioinformatics studies have made significant advances in the identification of diagnostic biomarkers for CKD (Ahmed et al., 2022; Wang et al., 2022). However, purely assessing the differences between CKD cases and normal controls is far from meeting the growing need for its complex pathological features, risk stratification for progressive end-stage renal disease, and high-risk clinical outcomes. Early characterization of this heterogeneity is an important step in developing an individualized follow-up strategy for patients with CKD (Zheng et al., 2021). Several studies have explored the importance of dysregulated signaling events in various renal and environmental cells, suggesting that immune cell infiltration patterns have predictive power for clinical guidance (Şenbabaoğlu et al., 2016). A comprehensive and integrated exploration of immune subtypes based on multiple transcriptome expression profiles may be a nice addition to the mechanisms of CKD progression (Li et al., 2022).

Therefore, in this study, we systematically examined the differentially expressed IRGs and immune profiles of renal tissues among normal and CKD individuals for the first time. The biological features, enrichment pathways and immune profiles among the two subtypes were further investigated by dividing 440 CKD patients into two immune-related subgroups through the expression profiles of 15 characterized IRGs. Predictive models disclosing patients with diverse molecular clusters were developed by combining several machine learning algorithms. Finally, we also investigated the correlation between the diagnostic value of prospective markers and clinical characteristics. We hope that a feature-based classification model for IRGs will reveal important molecular mechanisms underlying the role of immunity in CKD and provide for improved diagnosis, management and treatment of CKD.

## Materials and methods

### Data source

The CKD-associated microarray expression data were obtained from the Gene Expression Omnibus (GEO) database (https://www.ncbi.nlm.nih. gov/geo/). CKD datasets were searched using the following key terms: “CKD”, “human genome” and “chronic kidney disease” and screened according to the following criteria: (1) each dataset consisted of at least 30 samples; (2) the data tissue type is renal interstitial tissue; and (3) the original data is available in the GEO database and the type of experiment is microarray. On the basis of these criteria, the analysis included four CKD-related datasets GSE66494, GSE69438, GSE99325, and GSE104954 (Ju et al., 2015; Nakagawa et al., 2015; Shved et al., 2017; Grayson et al., 2018), of which the GSE66494 dataset contained 53 CKD and 8 normal samples; GSE69438 dataset had 42 CKD cases; GSE99325 dataset included 80 samples (CKD: normal = 171:4); and the ratio of CKD: normal was 174:21 in the GSE104954. A total of 440 CKD patients and 33 normal controls were obtained from the dataset, and all were renal interstitial tissue samples. GSE66494 was used as an external verification set.

**FIGURE 1:**
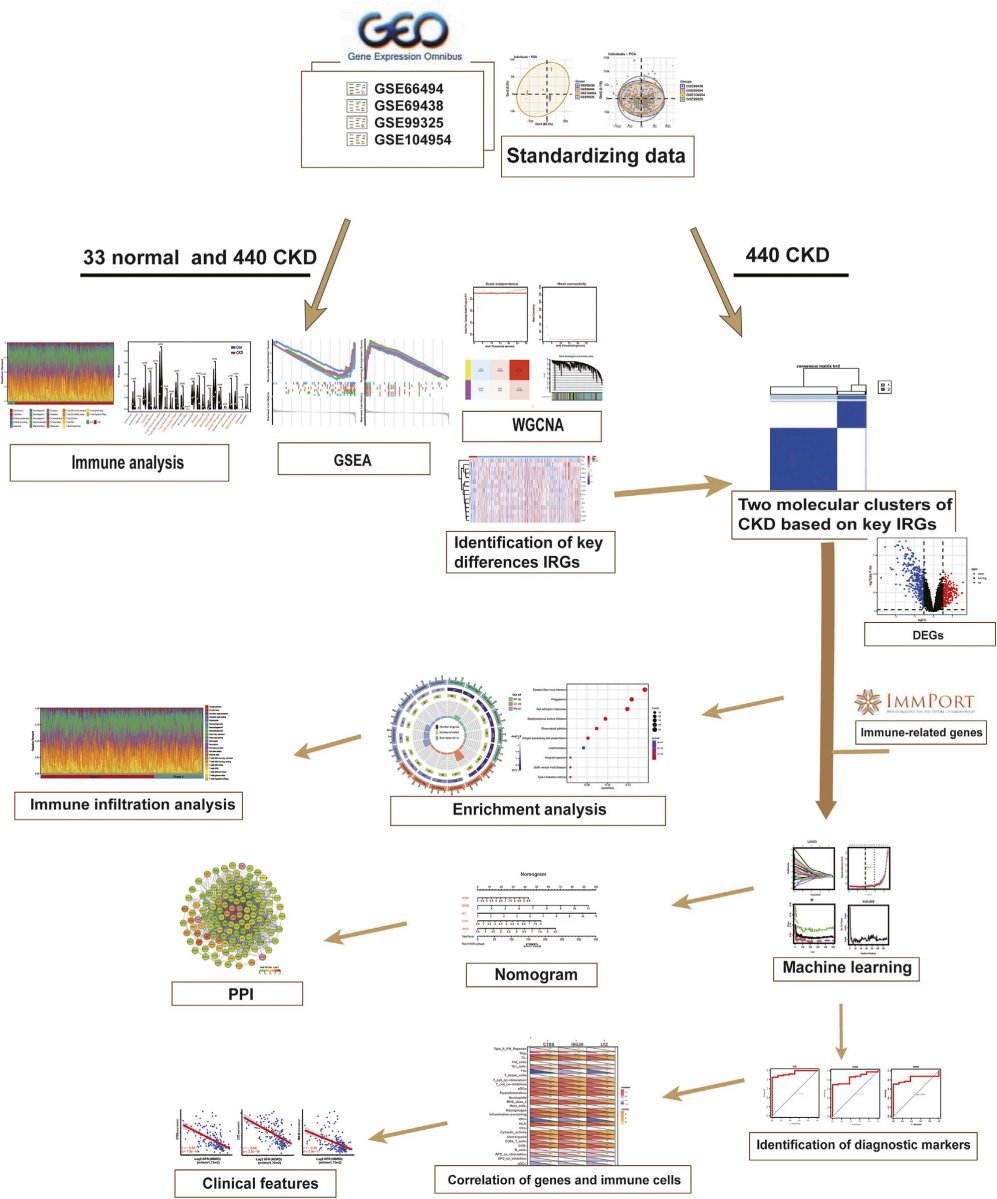
Work flowchart of the study.

These four raw datasets were batch corrected to eliminate batch effects for subsequent analysis using the “sva” R package (version 3.44) (Ritchie et al., 2015). Principal component analysis (PCA) was employed to visualize the distribution pattern across the samples. DEGs were screened by “limma” package (version 3.52.3), and *p* < 0.05, |log2(fold change, FC)| > 0.5 were considered statistically significant differences. After excluding duplicates, the IRGs were obtained from the Immunology Database and Analysis Portal (ImmPort) website (https://www.immport.org) (Bhattacharya et al., 2014). A total of 1793 genes were acquired for later research. The general flow chart of our study is presented in Figure 1.

### Weighted gene co-expression network analysis

Each sample’s immune gene set enrichment score was determined using gene set variance analysis (GSVA), and the R package “WGCNA” (version 1.71) was applied to build a score-based weighted gene co-expression network (Langfelder and Horvath, 2008). The optimal soft threshold was determined to convert the gene expression matrix into a weighted adjacency matrix and subsequently into a topological overlap matrix (TOM). Next, a hierarchical clustering method identified the modules. At last, Pearson correlation analysis was performed to assess the correlation among the three phenotypes of the immune score, normal control and CKD groups and the genes encompassed in each module.

### Consensus cluster analysis

Based on the key differential IRGs in the relevant modules obtained by WGCNA, a cluster analysis was performed using the ConsensusClusterPlus algorithm to identify potential subclusters of 440 CKD cases.

The cumulative distribution function (CDF) curve and consistent clustering score were utilized to determine the best k-value. In the meantime, PCA was applied to validate the classification of different subgroups.

### Functional and pathway enrichment analysis

DEGs between different immune-related subtypes were determined using the limma package with *p* < 0.05 and |log2 FC| > 0.5 as cut-off thresholds. The volcano map was mapped using the “ggplot2” R package (version 3.4.0). Meanwhile, Gene Ontology (GO), Kyoto Encyclopedia of Genes and Genomes (KEGG) enrichment analysis of DEGs were conducted utilizing the “clusterProfiler” package of R software (version 4.4.4). For Gene Set Enrichment (GSEA) analysis, the “c2.cp.kegg.v7.4.symbols.gmt” file was selected from the MSigDB online database (http://www.gsea-msigdb.org/gsea/msigdb). The first six significantly enriched pathway gene sets are shown. Adjusted *p* values less than 0.05 were determined to be significantly meaningful.

### PPI network construction

The use of the STRING database facilitates the development of Protein-Protein Interaction (PPI) networks. A list of differential IRGs was uploaded to the STRING database for identification and integration to construct PPI networks with a default composite score greater than 0.4. PPI network files were exported and re-visualised using Cytoscape 3.9.1 software.

### Immune cell infiltration analysis

Cell type identification was performed in the R software using the CIBERSORT method to assess the relative abundance of 22 immune cell infiltrates in every sample between different groups (Newman et al., 2015). Violin plots were plotted to visualise the different expression levels of 28 immune infiltrating cells. The association between immune cells and diagnostic markers was examined using Spearman correlation analysis. In addition, the immunological characteristics of the diagnostic markers were further analysed by “GSVA” based on the gene set of 29 immune-related responses (Bindea et al., 2013).

### Screening and validation of diagnostic markers for different immune subtypes of CKD

Three machine learning methods, LASSO logistic regression, SVM-RFE and Random Forest algorithms were used to select key differential genes between subtypes. LASSO analysis is a method of feature selection based on the “glmnet” R package (version 4.1-4) using 10-fold cross-validated turning/penalty parameters by reducing the dimensionality of high-dimensional data. SVM-RFE is a sequential backward selection algorithm that removes redundant features by ranking each feature with a score based on the maximum interval principle of SVM. SVM-RFE was applied to feature selection for ten-fold cross validation. The RF algorithm is an integrated approach to ranking the importance of immune-related genes through the “randomForest” package (version 4.7-1.1) in the R software. Specific genes are selected by obtaining the minimum error rate of the model. After creating a nomogram model with the “rms” R package (version 4.3-0), the constructed model’s clinical value and predictive power were assessed using calibration curves, decision curve analysis (DCA), and clinical impact curves (CIC).

The “pROC” program (version 1.18.0) was applied to perform receiver operating characteristic (ROC) analysis and generate area under the curve (AUC) values in order to identify key genes with high differential gene diagnostic efficiency obtained by screening as diagnostic markers.

### Analysis of the clinical relevance of biomarkers

Correlation between biomarkers and renal function was performed with the Internet Nephroseq v5 online database (http://v5.nephroseq.org).

### Statistical analysis

All statistical tests were implemented using the Rstudio software (version 3.6.3). Correlation analysis was achieved through Pearson’s analysis. Wilcoxon test was performed to determine the differences in immune scores among the two groups. Logistic regression algorithms were applied to build predictive models. Additionally, Independent sample t-tests were applied to compare the differential expression levels among the two groups. If not otherwise specified, *p* < 0.05 was deemed statistically relevant.

## Results

### Identification of core IRGs in CKD

**FIGURE 2:**
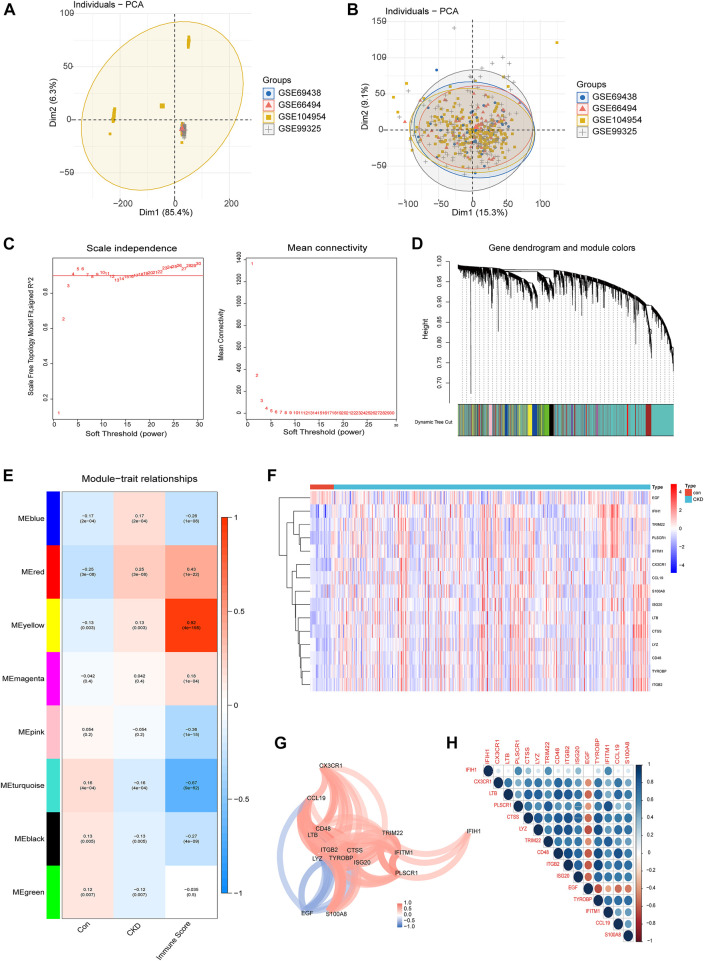
Identification of core IRGs in CKD. **(A, B)** PCA results of sample clustering before **(A)** and after **(B)** batch calibration. **(C)** Filtering of soft thresholds. **(D)** Cluster trees of co-expressing genes. **(E)** Division of gene modules, heat map of the relationship between building blocks and characteristics. **(F)** Representative heatmap of 15 differentially expressed IRGs. **(G)** Diagram of the gene relationship network for 15 IRGs with differential expression. **(H)** Representative correlation diagram of 15 differentially expressed IRGs. Blue is a positive correlation and red is a negative correlation.

The gene expression profiles of four GEO datasets (GSE66494,GSE69438, GSE99325 and GSE104954), including 33 normal controls and 440 kidney tissues from CKD patients, were acquired from the GEO database. All samples were clustered together after batch correction to remove batch effects and log normalization (Figures 2A, B), and the final integrated dataset was analyzed further.

Differentially expressed IRGs were identified by comparing the gene expression levels in renal tissues of normal control and CKD patients. Following that, we conducted GSVA enrichment analysis according to the expression profiles of all IRGs, and were able to determine the immune scores for each sample. The immunological score and sample grouping information were combined to create a scale-free network, with a soft threshold of 4. WGCNA discovered a total of 8 modules, each of which was labeled with a distinct color. Among them, the yellow module is most associated with the CKD immune score (Figures 2C–E). The yellow module contains 15 important differentially expressed IRGs, comprising fourteen upregulated and one downregulated genes (Figure 2F), and we visualized these 15 IRGs through gene relationship network plots to better analyze the interactions and interrelationships between genes (Figure 2G). In addition, Spearman correlation analysis elucidated the correlation patterns between these genes (Figure 2H). Among these IRGs, EGF was negatively associated with a majority of genes.

### Activation of the immune system in CKD patients

**FIGURE 3:**
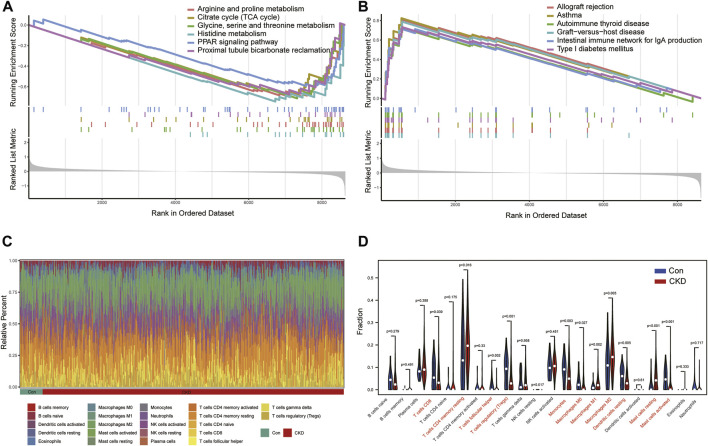
Analysis of GSEA enrichment and immunoinfiltration among the normal and CKD patients **(A)** Six enriched KEGG pathways in normal samples. **(B)** Six enriched KEGG pathways in CKD samples. **(C)** Barplot of the relative frequency of 22 different immune cell infiltration in normal and CKD samples. **(D)** Vioplot of the difference of 22 different immune cells in normal and CKD samples.

To better explore the potential functional and signaling enrichment pathways between control and CKD subjects, we performed GSEA enrichment analysis (Figures 3A, B). The results showed that metabolism-related pathways were concentrated in normal samples, whereas the allograft rejection, asthma, autoimmune thyroid disease, graft−versus−host disease, intestinal immune network for IgA production, T ype I diabetes mellitus were significantly enriched in the CKD group, suggesting that immune imbalance may be one of the main pathogenic mechanisms in the advancement of CKD.

Therefore, we further analyzed the differences in the proportions of 22 immune cell subtypes between the normal and disease states according to the CIBERSORT algorithm (Figure 3C), and the results showed that CKD patients had higher levels of T-cell CD4 memory resting, Macrophages M1, Macrophages M2, Mast cells resting, and the results confirmed that activation of the immune system is closely associated with the progression of CKD (Figure 3D).

### Identification of molecular clusters in CKD

**FIGURE 4:**
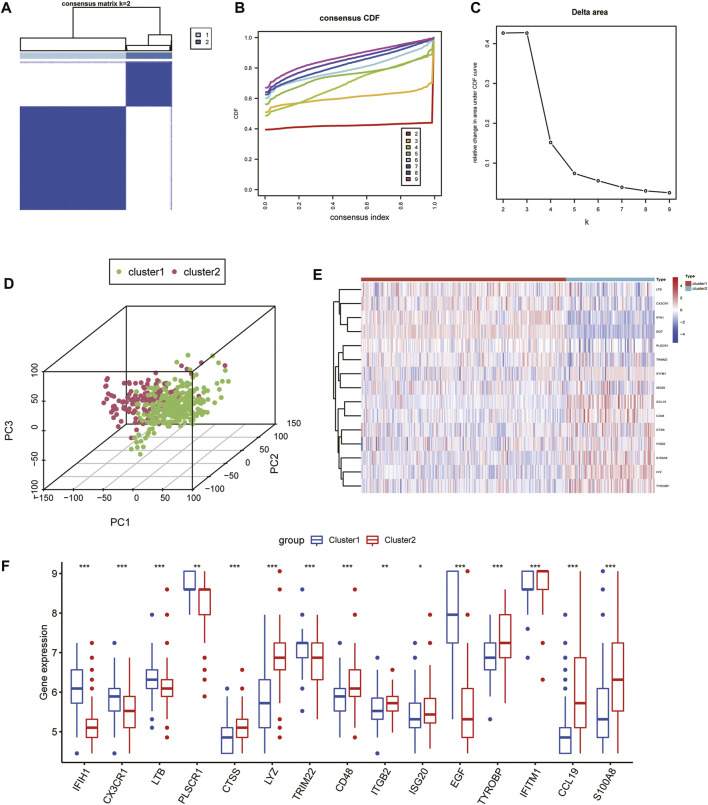
Unsupervised consensus clustering in CKD samples based on IRGs. **(A)** Consensus clustering matrix displaying the two CKD sample clusters with k = 2. **(B)** Cumulative distributive function for k = 2 to 9. **(C)** Delta graph displaying the change in the CDF curve’s area from k = 2 to 9. **(D)** PCA scatter plot based on the results of cluster analysis. **(E, F)** Heatmap **(E)** and box plots **(F)** of 15 IRGs among the two clusters. ***p < 0.001; **p < 0.01; and *p < 0.05.

To explore the role of IRGs in CKD clusters, we performed an unsupervised consensus cluster analysis on 440 CKD samples according to the expression of the 15 IRGs. The combined assessment of clinical significance and clustering effects showed that clustering was highly stable and reliable when k = 2 (Figure 4A), and the CDF curve of the consensus index was stable around 0.4 when the value of k was set to 2 (Figure 4B). When k = 2-9, the change in the delta region is shown in the CDF plot (Figure 4C). CKD samples were divided into cluster 1 with 307 samples and cluster 2 with 133 samples. Moreover, the principal component analysis (PCA) clustering results further confirmed that cluster 1 and cluster 2 were well differentiated (Figure 4D). To explore the molecular features between clusters, we used heat maps and box plots to depict the differential expression levels of 15 core IRGs between Cluster1 and Cluster2 (Figures 4E, F). Cluster1 exhibited significantly higher levels of IFIH1, CX3CR1, LTB, PLSCR1, TRIM22, and EGF, while Cluster2 had markedly enhanced expression levels of CTSS, LYZ, CD48, ITGB2, and TYROBP, IFITM1, CCL19, and S100A8.

### Identification of cluster-specific DEGs and functional annotation

**FIGURE 5:**
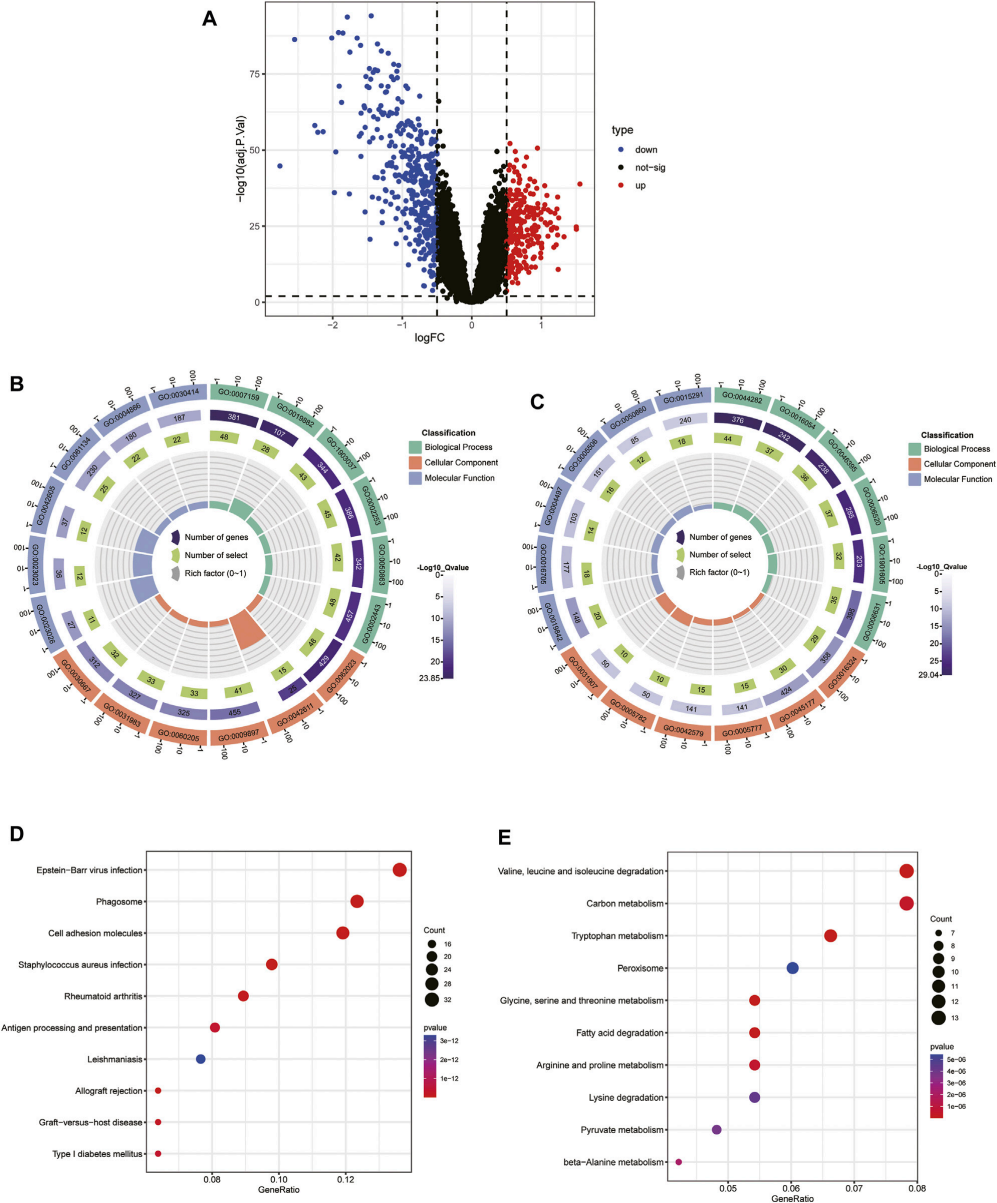
Identification of the DEGs and enrichment analysis between the two molecular subtypes.**(A)** Volcano plot of the DEGs among cluster one and 2. **(B, C)** GO analysis of upregulated DEGs in cluster 1 **(B)** and cluster 2 **(C)**. **(D, E)** KEGG analysis of the upregulated DEGs in cluster 1 **(D)** and cluster 2 **(E)**.

To explore the biological functional differences between the different subtypes, we first identified 634 DEGs. Among them, there were 246 upregulated genes and 388 downregulated genes identified in cluster one compared to cluster 2 (Figure 5A). GO functional enrichment analysis indicated that most of the upregulated genes in cluster 2 were enriched in defense processes (e.g., leukocyte cell-cell adhesion and regulation) and immune-related processes (e.g., antigen processing and presentation, activation of the immune response, regulation of T-cell activation) (Figure 5B), whereas metabolism-related signaling processes, biosynthesis, and stimulated response were upregulated in cluster 1 (Figure 5C). KEGG enrichment analysis indicated that immune and inflammation-related disease pathways such as infection and allograft rejection, rheumatoid arthritis, and graft-versus-host disease were important signaling pathways for Cluster 2 (Figure 5D), while metabolism-related pathways were mainly enriched on Cluster 1 (Figure 5E). These results reveal significant differences in enrichment pathways and biological functions among immune subgroups of CKD patients.

### Identification of two subtype-specific gene patterns by machine learning

**FIGURE 6:**
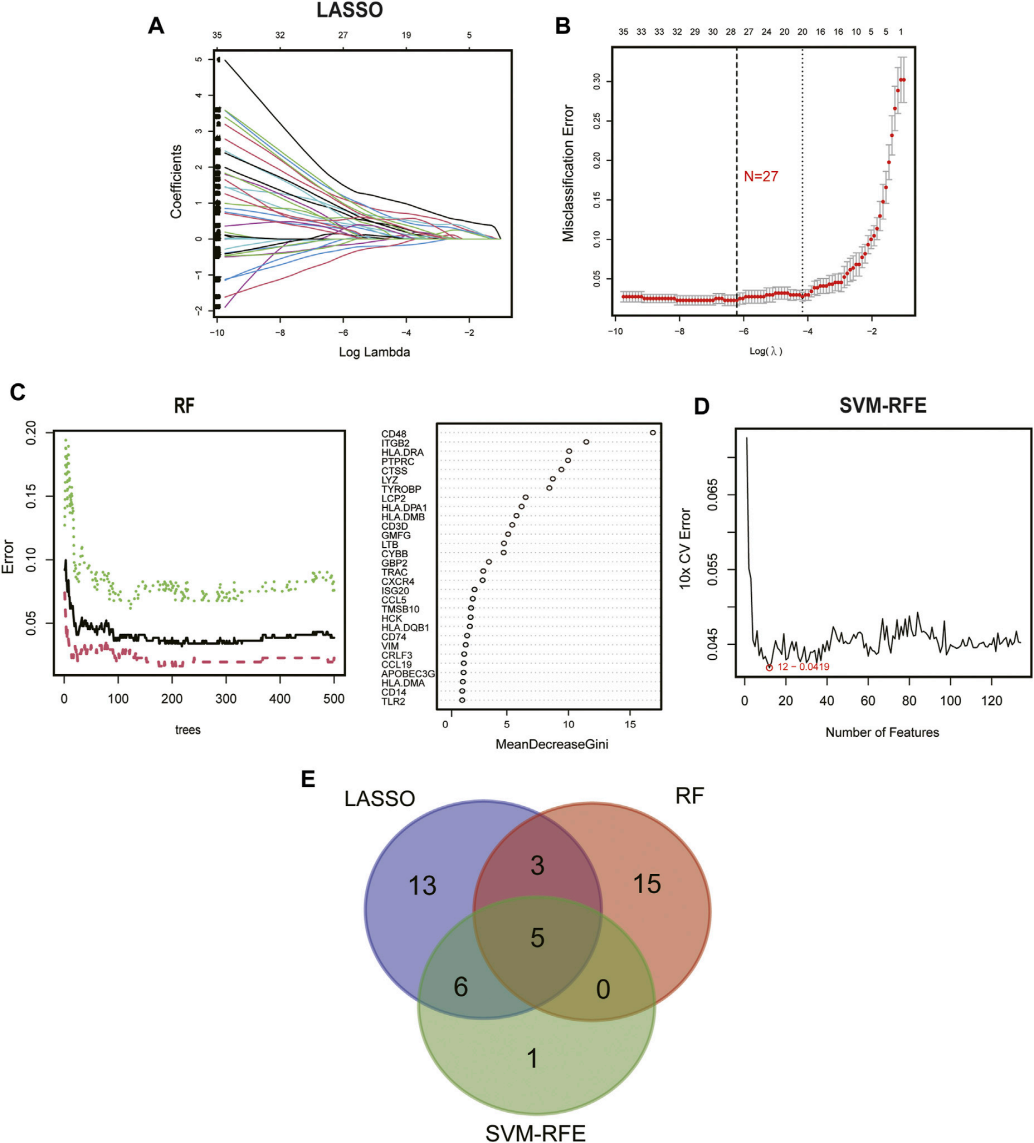
Identification of diagnostic biomarkers from subtypes using machine learning algorithms. **(A)** The LASSO coefficient profiles analysis. **(B)** Selecting the appropriate lambda value for a LASSO regression model. **(C)** Random forest trees constructed by cross-validation and gene ranking by importance score. **(D)** Estimation of 10-fold cross-validation error using SVM-RFE method. **(E)** Venn plot illustrating the key genes among LASSO, RF, and SVM-RFE.

To further elucidate the role of immune in the heterogeneity of CKD patients, we applied machine learning methods to screen subtype-specific potential genes for constructing prediction models. First, using a LASSO regression model with 10-fold cross-validation, we proved the optimal value of λ and screened 27 potential candidate biomarkers in IRGs for inter-subtype differences (Figures 6A, B). The RF algorithm ranked genes according to the calculated importance of each gene and identified 23 valid predictors (Figure 6C). By using the SVM-RFE algorithm, 12 genes were extracted as potential key genes (Figure 6D). The crossover of the three algorithms was visualized by Venn diagram and five genes (CD48, CTSS, ITGB2, LYZ, and ISG20) were identified for final validation (Figure 6E).

**FIGURE 7:**
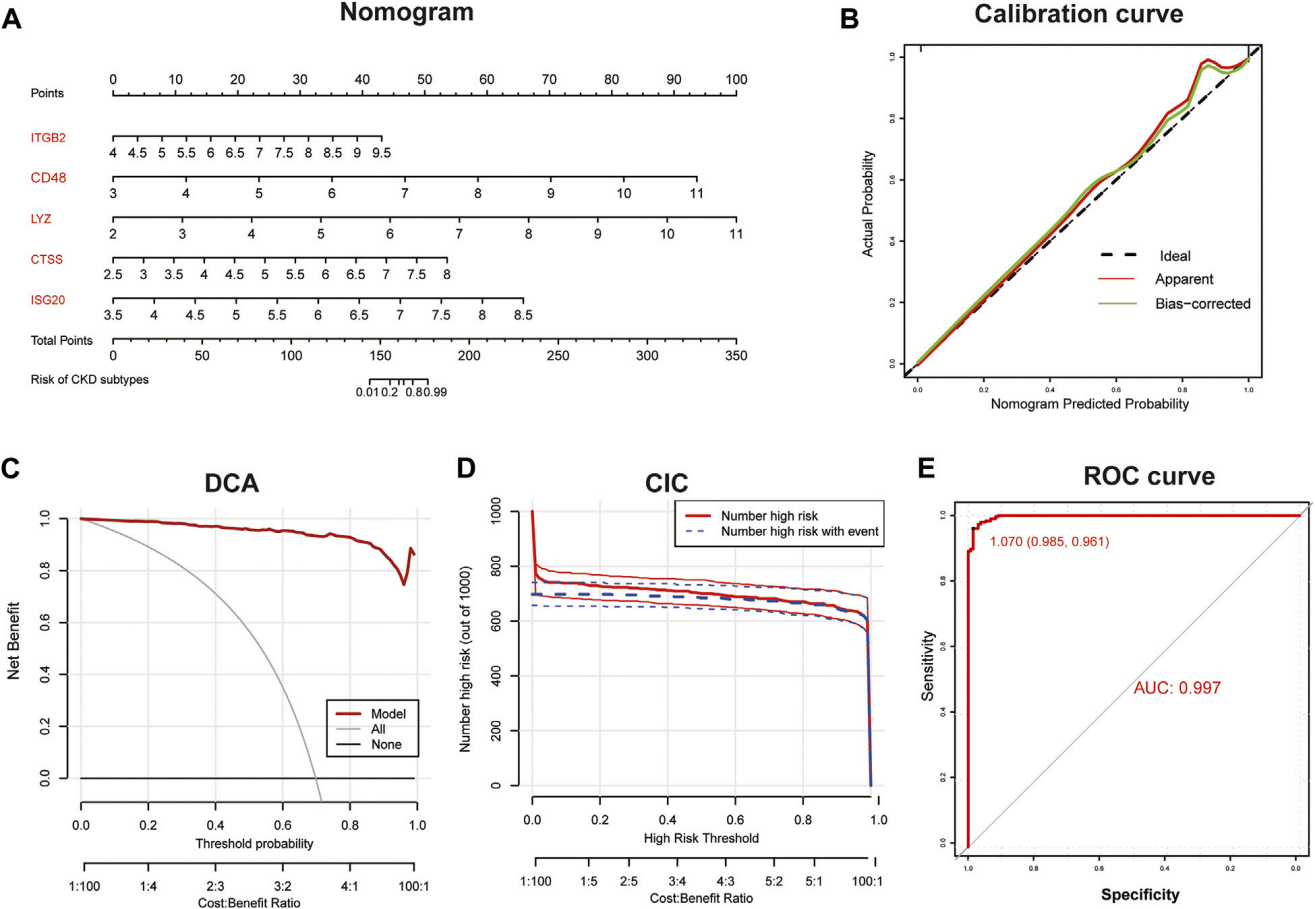
Construction of diagnostic Nomogram model. **(A)** Nomogram for predicting risk of CKD subtypes based on 5 key IRGs. **(B, C)** Calibration curve **(B)** and DCA **(C)** to estimate the predictive efficiency of the Nomogram model. **(D)** Clinical impact curves (CIC) to estimate the clinical validity of Nomogram model based on DCA curve. **(E)** ROC curves to evaluate the discrimination ability.

Subsequently, we constructed a diagnostic nomogram model to assess the risk of immune clustering in 440 patients with CKD (Figure 7A). The calibration curve showed that there was a small difference between the actual risk and the anticipated risk, indicating that the Nomogram model has great accuracy in predicting immune subtypes (Figure 7B). According to DCA, the values of the “model” curve of the gene of interest in the nomogram model are greater than the gray curve, suggesting a potential clinical benefit for patients within a threshold risk probability range of 0–1 (Figure 7C). Clinical Impact Curves (CIC) based on DCA showed that the nomogram model can be a sound basis for clinical decision making (Figure 7D). In addition, the model displayed a remarkably high AUC value (0.997), confirming the outstanding predictive properties.

### PPI network construction of immune-related DEGs among subtypes

**FIGURE 8:**
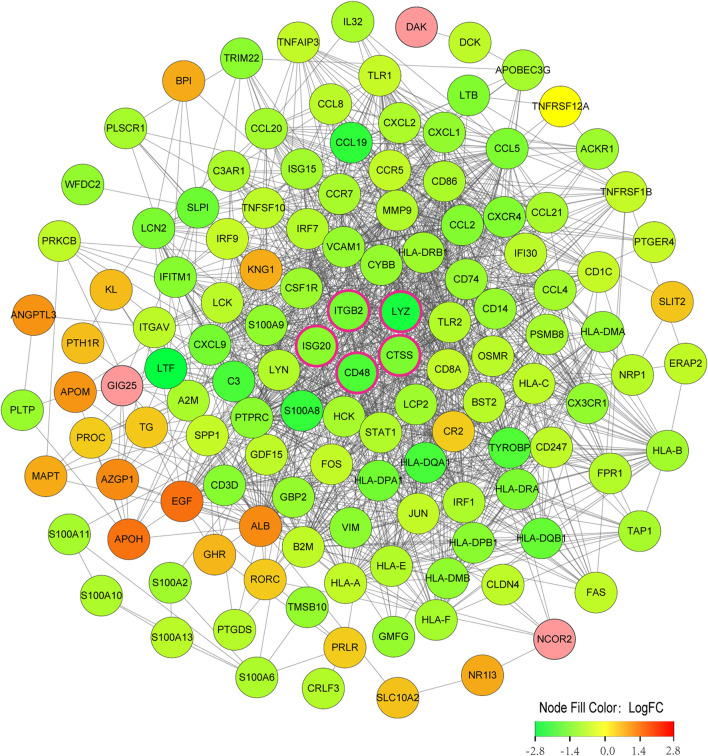
PPI network construction of immune-related DEGs among subtypes. Nodes with rose-colored outlines represent key candidate genes, green nodes (negative logFC) represent genes upregulated in Cluster1, and red nodes (positive logFC) represent genes upregulated in Cluster2. FC, fold change.

The 134 immune-related DEGs between subtypes were imported into STRING database, and the acquired data were introduced into Cytoscape to build a PPI network (Figure 8). A total of 130 nodes and 1,369 edges were obtained. Supplementary Table S1 summarizes the topological features of the first 30 nodes in the PPI network. After calculation, the degrees of five key candidate genes were CD48 (24°), CTSS (32°), ITGB2 (47°), LYZ (22°) and ISG20 (19°). Interestingly, all five key genes were upregulated in Cluster2 compared to Cluster1.

### Analysis of the diagnostic value of key candidate biomarkers

**FIGURE 9:**
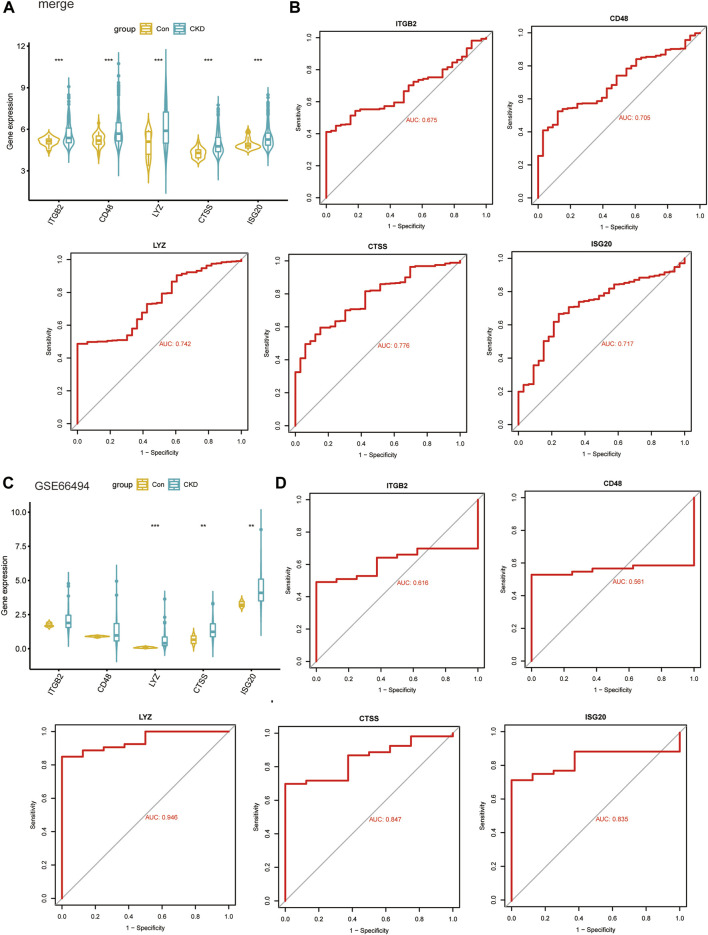
Validate clinical diagnostic capabilities of key biomarkers. **(A, B)** Expression levels **(A)** and diagnostic potency **(B)** of key genes in the models in the four combined datasets. **(C, D)** Expression levels **(C)** and diagnostic potency **(D)** of key genes in the models in the dataset GSE66494.

By using box plots, we evaluated the expression levels of 5 key candidate genes in CKD and normal sample. Figure 9A shows that the expression of all five genes in the CKD group was substantially higher than that in the normal controls. Subsequently, the expression levels of these five key genes were further validated in the dataset GSE66494, and the results showed that they had similar expression patterns (Figure 9C). Plotting the ROC curves of key genes allowed researchers to evaluate how well they performed in predicting illness samples. When we analyzed the AUC values of five important genes, four of them had values > 0.7, while the ITGB2 gene had an AUC value of 0.675, indicating that these genes were successful at differentiating between CKD and normal samples (Figure 9B). To validate their clinical efficacy, the diagnostic value of the five key genes mentioned above was further verified in the GSE66494 dataset. The results showed that CTSS, ISG20 and LYZ genes with AUC >0.8 had high diagnostic value (Figure 9D).

### Immune infiltration characteristics between the two subtypes

**FIGURE 10:**
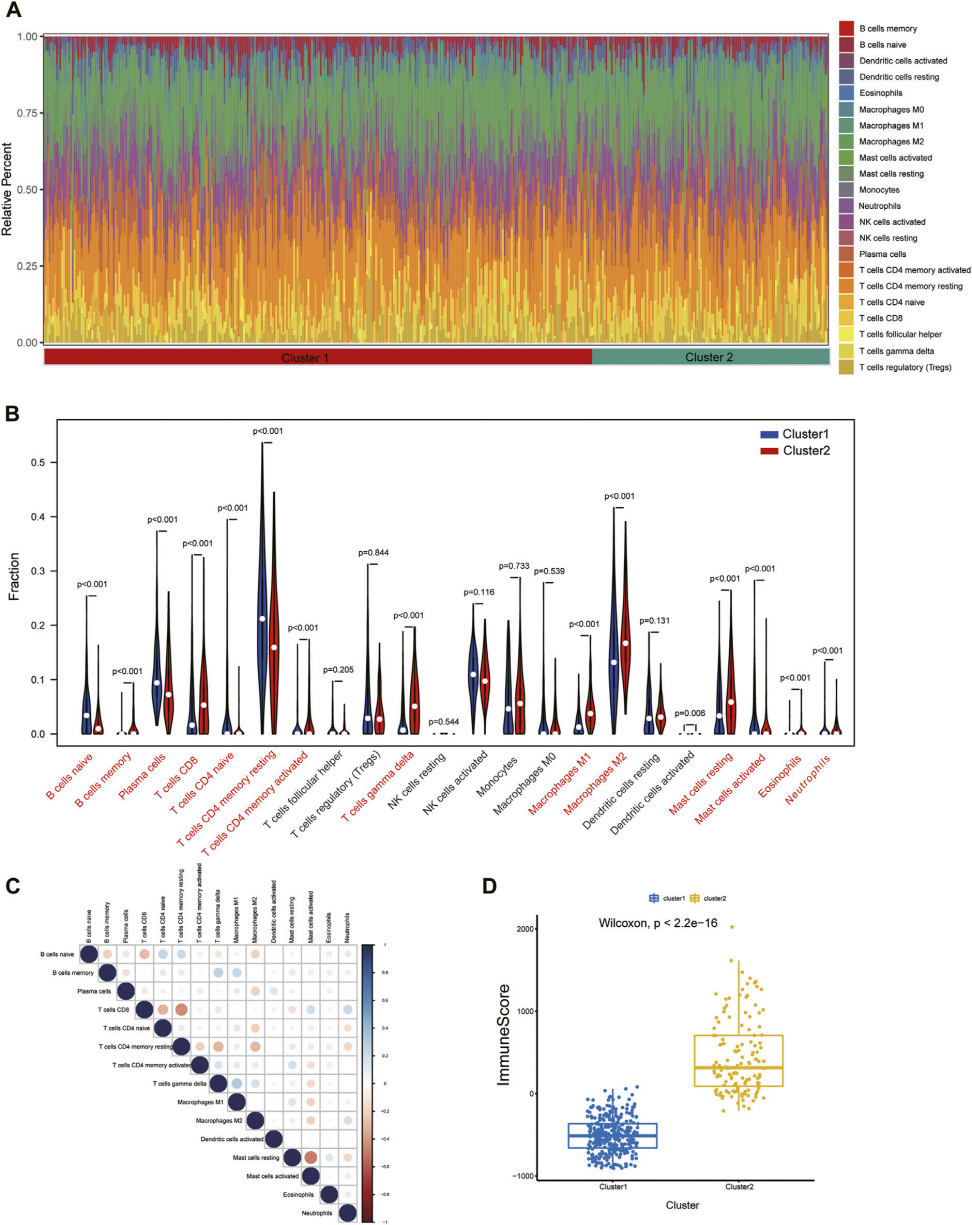
Immune cell infiltration differences among clusters. **(A)** Immune microenvironment of cluster1 and cluster2. **(B)** Box plots showing variation in immune cell infiltration among clusters. **(C)** Relevance heatmap of immune cells with disparities. **(D)** Estimated Immunoscore among subtypes.

To further assess the immune landscape between the two clusters, we quantified the level of 22 immune cell infiltrates in each patient sample using the CIBERSORT method (Figure 10A). Our results revealed that the proportions of naive B-cell, plasma cells, naive CD4 T-cell, memory resting CD4 T-cell, and activated mast cells were markedly higher in Cluster1, while Cluser2 exhibited a greater abundance of CD8 T-cell, T-cell gamma delta, M1 and M2 macrophages, and resting mast cells (Figure 10B). These differences suggest that there is abundant immune cell heterogeneity within renal tissue during disease progression. Meanwhile, a definite correlation was observed among immune cells of various infiltration degrees, with resting mast cells and memory resting CD4 T-cell were negatively correlated with most immune cells (Figure 10C). Additionally, we thoroughly compared the immune scores of both clusters and discovered that the Cluster2 had higher scores than the Cluster1 group, indicating an elevated level of immune cell infiltration in Cluster2 (Figure 10D).

**FIGURE 11:**
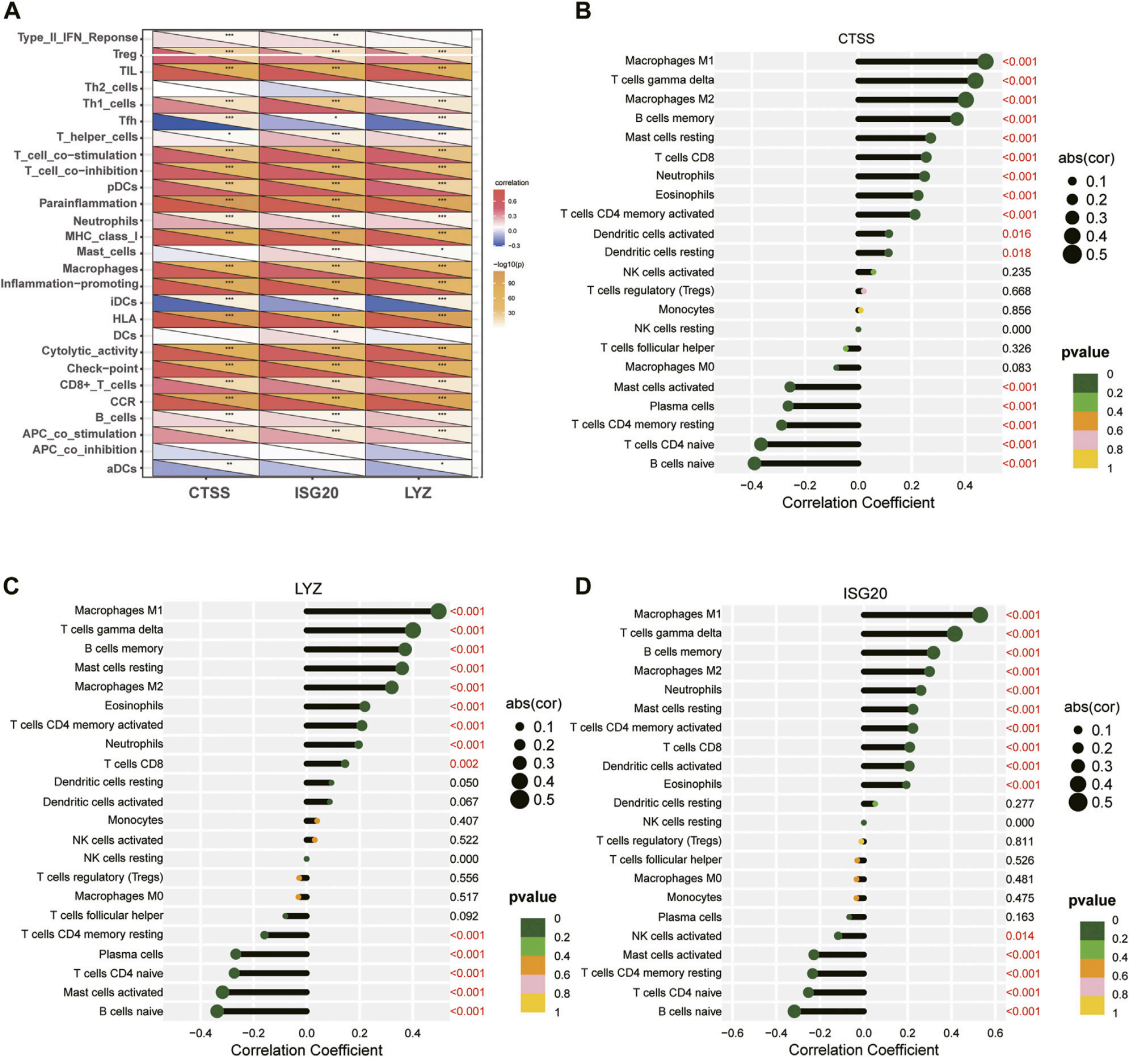
Relationship between three key diagnostic biomarkers and immune cell infiltration. **(A)** Visualization of immune cells or pathways in relation to three biomarkers by GSVA. **(B–D)** Correlation between immune cell infiltration and **(B)** CTSS, **(C)** LYZ, **(D)** ISG20 gene expression. ***p < 0.001; **p < 0.01; and*p < 0.05.

The correlation of three key diagnostic biomarkers with immune cell infiltration was explored based on the results of “GSVA” and “CIBERSORT”. The correlation results of GSVA showed that regulatory T-cell (Tregs), helper T (Th) one cells, plasmacytoid dendritic cells (pDCs), neutrophils, macrophages, CD8^+^ T-cell, and B-cell were significantly positively associated with CTSS, ISG20, and LYZ, while T-follicular helper (Tfh) and immature dendritic cells (iDCs) were highly negatively associated with these three genes. In addition, in terms of immune function, the activities of T-cell costimulation, T-cell coinhibition, parainflammation, major histocompatibility complex (MHC) class I, inflammation−promoting, human leukocyte antigen (HLA), check−point, cytolytic-activity, chemokine C-C-Motif receptor (CCR), and antigen-presenting cells (APC) costimulation were significantly positively correlated with the expression of three genes (Figure 11A).

**FIGURE 12:**
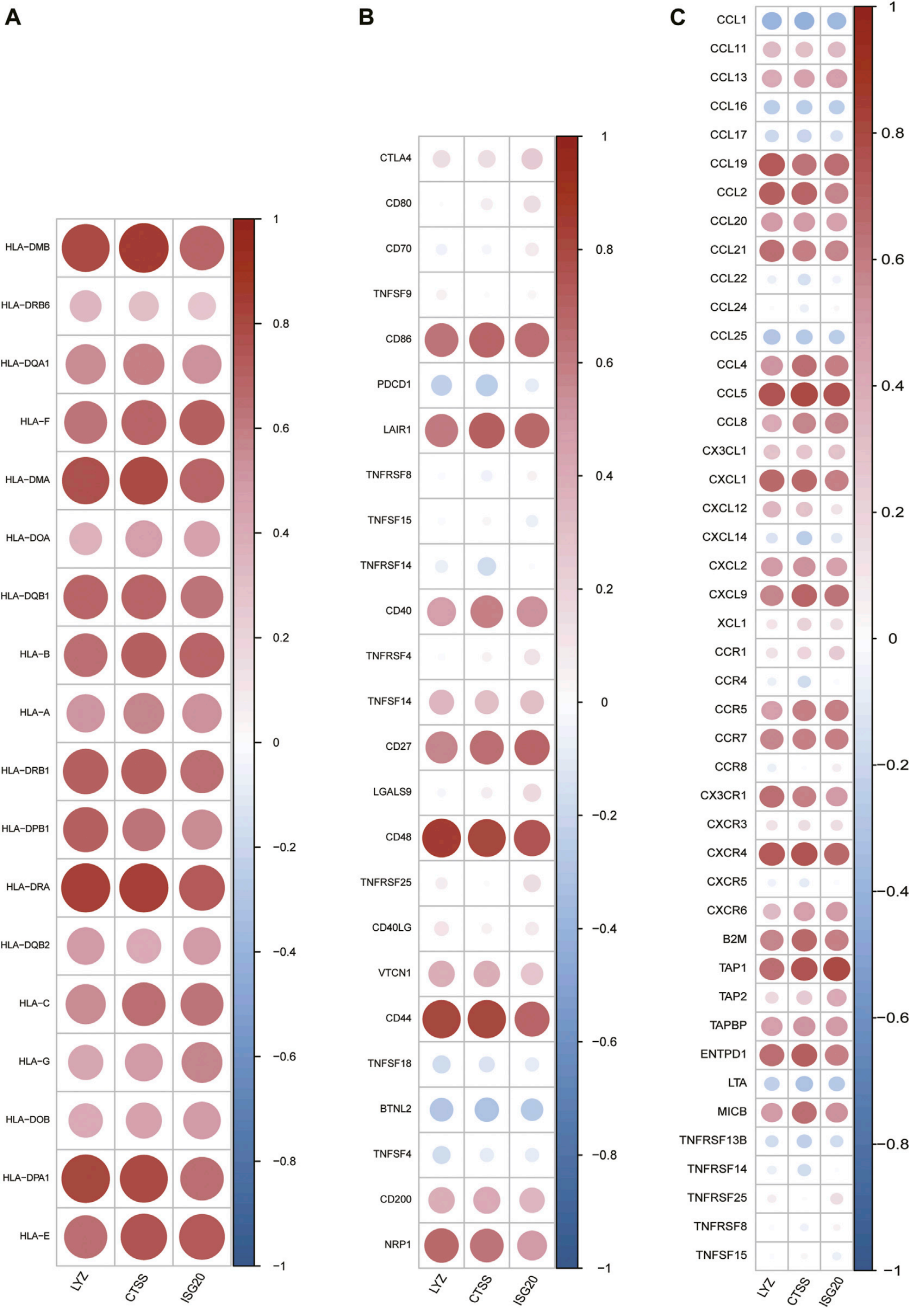
Correlation of IRG-DEGs with immune-related genes. **(A–C)** Correlation of IRG-DEGs with HLA **(A)**, immune checkpoint genes **(B)**, and immune receptor genes **(C)**.

Meanwhile, spearman correlation analysis indicated that CTSS, ISG20, and LYZ were all positively correlated with T-cell gamma delta, M1 Macrophages, M2 Macrophages, and memoryB cells, and negatively correlated with naive B-cell, memory resting CD4 T-cell, naive CD4 T-cell, and activated mast cells, confirming that these genes play an important role in differentiating subtypes (Figures 11B–D). In addition, we explored the correlation of IRG-DEGs with immune checkpoint genes, HLA, and immune receptor genes and found positive correlations with most of them (Figure 12).

### Expression profiles and clinical relevance of biomarkers

**FIGURE 13:**
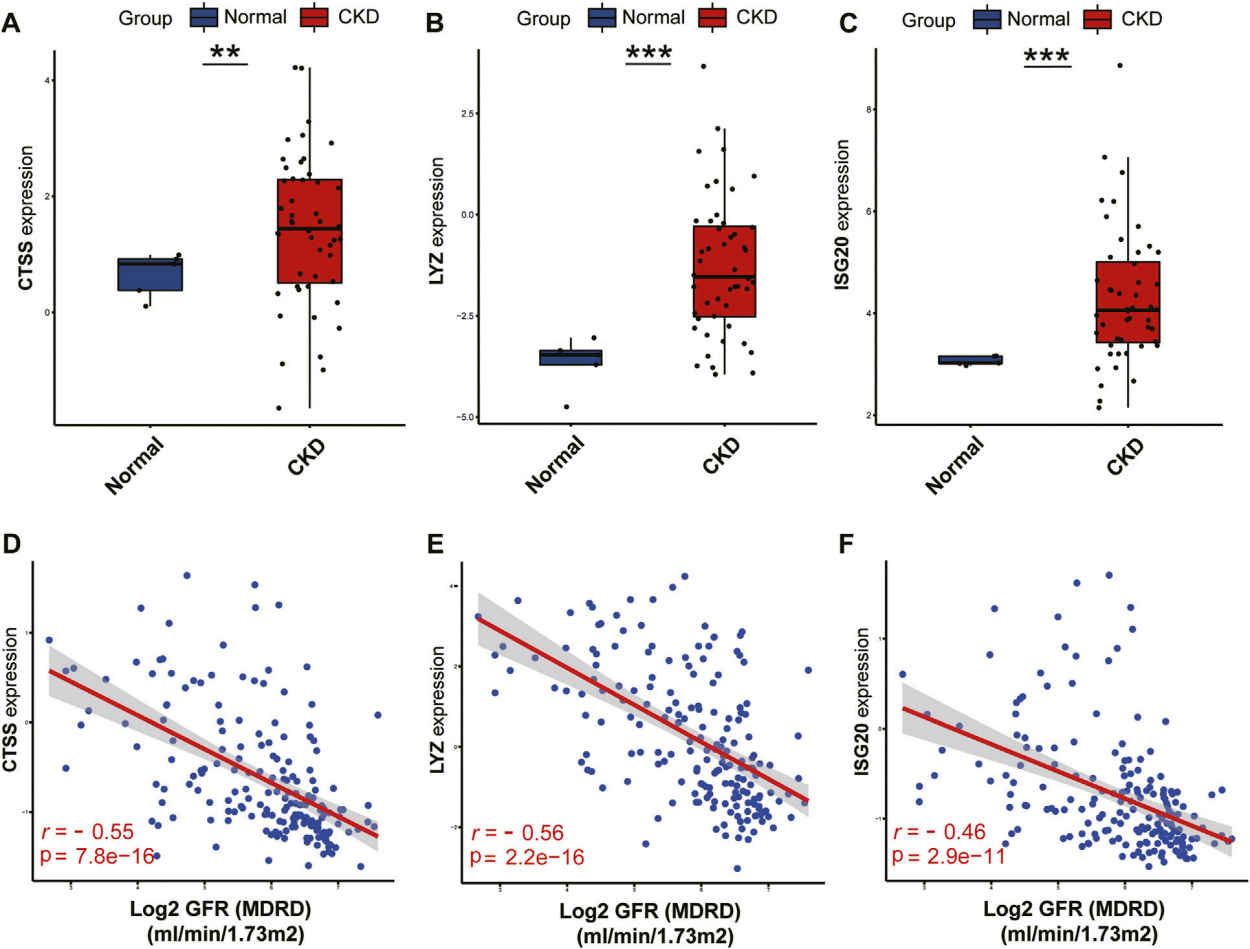
Validation of the three identified biomarkers and clinicality analysis.**(A–C)** Expression patterns of identified biomarkers CTSS **(A)**, LYZ **(B)**, ISG20 **(C)**. **(D–F)** Correlation between the expression of biomarkers CTSS **(D)**, LYZ **(E)**, ISG20 **(F)** and renal function indicators. ***p < 0.001; **p < 0.01; and *p < 0.05.

On the basis of the Nephroseq v5 online tool, we further confirmed the expression profiles of three potential diagnostic markers CTSS, LYZ and ISG20 in the renal tissues of CKD patients. The results displayed that the expression of all three candidate markers was markedly upregulated in CKD kidney tissues compared with normal kidney tissues (Figures 13A–C). In addition, correlation analysis showed that the expression of all three potential markers in CKD renal interstitial tissue was negatively correlated with glomerular filtration rate (GFR) (Figures 13D–F).

## Discussion

Chronic kidney disease affects 11.7%–15.1% of the global population and is one of the world’s most serious health problems (Lv and Zhang, 2019). Regardless of the potential cause, CKD progresses gently and contributes to irreversible nephron loss, end-stage renal disease, and increased mortality. The differential phenotypes of CKD are not always well known in clinical practice due to the presence of marked heterogeneity in pathogenic processes, risk factors, pathological changes, and outcomes, and thus their overall treatment outcomes are extremely limited. Previous studies have emphasized the development of tools for characterizing this potential heterogeneity with different phenotypic data (Zheng et al., 2021), but no in-depth analysis of CKD subgroup-specific biomarker data has been performed. The addition of distinguishing CKD clusters at the molecular level will undoubtedly increase our comprehension of CKD heterogeneity and is important for guiding individualized treatment of CKD. Furthermore, despite significant efforts to explore new targets for CKD, the current knowledge appears to be inadequate and there is still an urgent need for potential biomarkers with high specificity and sensitivity.

Immunomodulation has been extensively studied due to its close relationship with CKD. As previously mentioned, CKD is a chronic and confusing process that affects a variety of normal metabolic functions and kinase-mediated pathways. Chronic inflammation underlies one of the major pathogenic mechanisms of chronic kidney disease, a process that can be characterized as a complicated interaction among renal cells and resident immune cells, such as dendritic cells and macrophages, and the recruitment of circulating monocytes, neutrophils and lymphocytes (Andrade-Oliveira et al., 2019). With further exploration of the mechanisms of monocytes (Heine et al., 2012), macrophages (Guiteras et al., 2016), mast cells (Holdsworth and Summers, 2008), T-cell and dendritic cells (Weisheit et al., 2015) in CKD revealed that they are closely related with the progression and development of CKD. Characterizing the molecular role of immune cell infiltration into CKD is crucial, and immunogene features may be a prognostic or predictive factor for CKD.

In this study, we first comprehensively analysed the expression profiles of IRGs in normal and CKD patient‘s renal interstitial tissue samples and identified 15 important core genes that may be involved in the immune-related pathological processes of CKD. Most of these IRGs were higher in CKD patients than in normal controls, suggesting that immune regulation may play a key role in the pathogenesis of CKD. In addition, we explored the differences in immune cell levels between them and found that CKD patients showed higher levels of T-cell, macrophages and mast cells, which is consistent with earlier studies (Zeisberg and Neilson, 2010; Lee et al., 2020).

Subsequently, we investigated for the first time the identification of two distinct immune-related clusters based on central IRGs. Cluster 2 showed higher immune scores and levels of immune infiltration than Cluster 1. GO enrichment analysis of the two clusters revealed that leukocyte cell adhesion and regulation, as well as immune-related processes (e.g., antigen processing and presentation, immune response activation, T-cell activation regulation) were significantly different immune processes. The buildup of interstitial leukocytes is a feature of numerous CKD subtypes. Leukocyte adhesion is a key link in the pro-inflammatory, pro-apoptotic, and pro-fibrotic mediators produced by interstitial leukocyte infiltration, thereby contributing to the progression of CKD (Anders et al., 2006). Locally secreted chemokines play a key role in the progression of CKD by mediating the recruitment of inflammatory leukocytes and activation of immune cells (Anders et al., 2006). Additionally, enrichment analysis suggests that clustering distinguishes CKD patients with different levels of immune response and immune activation. KEGG enrichment analysis reveals distinction between immunometabolic disorder pathways and abnormal immune diseases in CKD subtypes.

Given that immune molecular features may be key regulators of individual heterogeneity in CKD. Based on three machine learning algorithms, we next evaluated immune-related DEGs between the two clusters and selected five important immune-related genes (CD48, CTSS, LYZ, ITGB2, and ISG20) to predict subtypes of CKD patients. The CD48 molecule is a glycosylphosphatidylinositol (GPI)-anchored cell surface protein of the CD2 family of molecules, and through its interaction with the ligands CD2 and CD244, it contributes to a variety of immune processes, including immune prominence of tissues, cell adhesion and co-stimulation, regulation of target cell lysis, and promotion of T-cell activation (Mcardel et al., 2016). ISG20 (interferon stimulated exonuclease gene 20) is an RNA nucleic acid exonuclease that stimulates the progression of a variety of tumors (Gao et al., 2019), and enhanced ISG20 expression is correlated with greater infiltration of monocyte-derived macrophages and neutrophils and suppresses adaptive immune responses (Gao et al., 2019). The expression of ISG20 was significantly elevated in renal fibrosis compared to normal samples, and *in vitro* knockdown of ISG20 significantly inhibited fibrotic protein expression in HK-2 (Sun et al., 2022).

As a member of the cysteine protease family, CTSS (cathepsin S) levels are elevated in a variety of diseases and are strongly associated with diseases such as IgA nephropathy, diabetes mellitus and atherosclerosis (Liu et al., 2006; Zhao et al., 2021). According to a recent study, macrophage-derived CTSS may hasten endothelial damage and nephrosclerosis in diabetic nephropathy (Kumar Vr et al., 2016). In addition, CTSS mediates the TGF-β/smad signalling pathway to promote extracellular matrix deposition and epithelial-mesenchymal transition (EMT) in the kidney (Yao et al., 2019). ITGB2 is capable of encoding integrin β-chains that binds to other α-chains to form distinct integrin heterodimers. It is involved in cell surface-mediated signalling and immune responses, and functions to promote leukocyte adhesion, extravasation and extracellular matrix remodelling (Tan, 2012; Boguslawska et al., 2016). LYZ (lysozyme) is an innate immune protein released from neutrophil and macrophage granules (Faust et al., 2000), which has antimicrobial properties and is involved in immune regulation, inflammatory signalling, vasodilation and myocardial inhibition (Mink et al., 2008). A recent study indicated a significant association of LYZ with all CKD stages, eGFR and survival (Makridakis et al., 2020). LYZ may be a promising therapeutic target for chronic kidney disease. Subsequently, we constructed a nomogram model for the diagnosis of CKD subtype based on the 5 gene and found that the model had significant predictive power, suggesting the value of this prediction model in clinical application. In other words, the model can provide clinicians with a trial strategy to pre-emptively identify patients at high risk of CKD immunisation and to develop early interventions to slow the trajectory of renal decline in patients.Notably, all of these biomarkers were overexpressed in the CKD samples compared to controls. The potential diagnostic value of these markers was also further analysed and CTSS, LYZ, and ISG20 were found to be highly accurate in differentiating CKD from normal tissue and to correlate closely with renal function.

For the immune infiltration results between clusters, we discovered that Cluster 1 significantly infiltrated cells were naive B-cell, plasma cells, naive CD4 T-cell, memory resting CD4 T-cell, and activated mast cells, whereas Cluster 2 had a higher abundance of CD8 T-cell, T-cell gamma delta, and M1 and M2 macrophages. Studies between immune metabolism and naive B-cell and plasma cells have been extensively explored. When naive B-cell are activated, oxidative phosphorylation (OXPHOS), TCA cycling, and nucleotide biosynthesis increas (Waters et al., 2018). Plasma cells are able to rely on glutamine and long-chain fatty acids as substrates for oxidative metabolism to provide underlying oxidative phosphorylation (Raza and Clarke, 2021). Whether these immune cells are associated with the enhanced metabolic pathway in cluster 1 is unclear. Mast cells and CD4 T-cell exert a role in regulating inflammatory cell infiltration, but their role in CKD should be interpreted with caution (Kim et al., 2009; Tapmeier et al., 2010). Depletion of naive T-cell and CD4^+^ central memory cells is associated with progressive renal function decline (Litjens et al., 2006). γδ T-cell promote renal interstitial fibrosis (Law et al., 2019). Macrophages, as important heterogeneous cells of the innate immune system, can exhibit different phenotypes in response to the local microenvironment. Most forms of renal inflammation are characterized by M1 macrophage infiltration in the early stages, but M2 macrophage infiltration predominates in the chronic phase, and polarization of macrophages is noted during CKD (Lee et al., 2020). It is reasonable to expect that elevated M1 and M2 macrophages are observed in CKD. Overall, these results suggest that subtype 2 has higher levels of immune infiltrating cells that promote CKD progression than subtype 1, representing a worse prognosis for CKD. Finally, we evaluated the correlation between the biomarkers CTSS, LYZ and ISG20 and infiltrating immune cells. CTSS, ISG20, and LYZ were all positively correlated with T-cell gamma delta, M1 and M2 macrophages, and memory B-cell and negatively correlated with naive B-cell, naive CD4 T-cell, memory resting CD4 T-cell, and naive CD4 T-cell, confirming that these genes play an important role in differentiating subtypes. Furthermore, these genes are positively correlated with T-cell costimulation, T-cell coinhibition, parainflammation, MHC class I, inflammation-promoting genes, cytolytic activity, human leukocyte antigen (HLA), checkpoint activation, chemokine C-C-motif receptor (CCR), and antigen-presenting cells (APC) costimulation. Overall, a deeper understanding of immune cell-biomarker correlations may provide new insights into immune mechanisms, and targeting CTSS, LYZ, and ISG20 to improve abnormal immune status may be a promising method for treating CKD.

We collected sufficient samples and used comprehensive bioinformatics analysis methods, such as WGCNA and machine learning, which made this study more comprehensive and reliable. However, some limitations of our study have to be acknowledged. First, under long-term chronic kidney disease pathophysiological conditions, the gene expression patterns of certain immune cell types may be influenced and adjusted due to a number of immunologic or non-immunologic complex pathogenetic factors. Further work exploring this differential variation is necessary. Secondly, our study mainly focused on interstitial renal data and a parallel comparison of glomerular and tubulointerstitial data seems to be better convincing and reliable. Furthermore, it is necessary to collect additional important clinical information such as age, gender, stage, prognosis, etc. To further verify the diagnostic properties of the model in predicting CKD clusters as well as to adjust the clinical prediction curves. Finally, this study is based on published data, and although external datasets were applied for validation, more experiments are needed to demonstrate the biological function of the studied biomarkers.

## Conclusion

In summary, this study identified two immune-related clusters in CKD patients by unsupervised clustering and used to elucidate the biological functions between different subtypes using a bioinformatics analysis approach.A diagnostic model based on five characteristic IRGs (CD48, CTSS, LYZ, ITGB2, and ISG20) identified by three machine algorithms was shown to have good ability to discriminate between subtypes. CTSS, LYZ, and ITGB2 were demonstrated to be promising diagnostic biomarkers for CKD by using external datasets, and they all showed a negative correlation with GFR. Immune cell infiltration is heterogeneous among subtypes, and CTSS, LYZ, and ITGB2 are closely associated with multiple immune cells. Our study preliminarily explored the underlying immunomolecular mechanisms leading to CKD heterogeneity, and these results may provide new insights to explore the pathophysiology and prognostic heterogeneity of CKD patients.

## Data Availability

The original contributions presented in the study are included in the article/Supplementary Material, further inquiries can be directed to the corresponding author.
