# Supplementary material for: Identification of immune-related molecular clusters and diagnostic markers in chronic kidney disease based on cluster analysis
Source: Front Genet. 2023 Feb 6;14:1111976. doi: 10.3389/fgene.2023.1111976 (PMC9939663; doi:10.3389/fgene.2023.1111976)
Supplement: Supplementary file 1 [file Table1.DOCX]

**SUPPLEMENTARY TABLE 1**Topological analysis results by degree-the first 30 proteins.

| Name | Description | Degree | Betweenness |
| --- | --- | --- | --- |
| CD8A  PTPRC  CD86  STAT1  TLR2  ALB  CCL2  CCL5  B2M  CCR5  ITGB2  MMP9  CXCR4  CCL4  CSF1R  CD74  TYROBP  VCAM1  HLA-A  HLA-E  CXCL9  HLA-DRA  IRF1  CCR7  IRF7  JUN  CXCL1  HLA-B  HLA-C  CYBB | CD8a molecule  protein tyrosine phosphatase receptor type C  CD86 molecule  signal transducer and activator of transcription 1  toll like receptor 2  albumin  C-C motif chemokine ligand 2  C-C motif chemokine ligand 5  beta-2-microglobulin  C-C motif chemokine receptor 5  integrin subunit beta 2  matrix metallopeptidase 9  C-X-C motif chemokine receptor 4  C-C motif chemokine ligand 4  colony stimulating factor 1 receptor  CD74 molecule  TYRO protein tyrosine kinase-binding protein  vascular cell adhesion molecule 1  major histocompatibility complex, class I, A  major histocompatibility complex, class I, E  C-X-C motif chemokine ligand 9  major histocompatibility complex, class II, DR alpha  interferon regulatory factor 1  C-C motif chemokine receptor 7  interferon regulatory factor 7  Jun proto-oncogene  C-X-C motif chemokine ligand 1  major histocompatibility complex, class I, B  major histocompatibility complex, class I, C  cytochrome b-245 beta chain | 70  67  61  56  53  52  51  50  50  49  47  46  45  44  42  41  40  40  37  37  37  36  36  36  35  35  35  33  33  33 | 1121.26253  906.67844  451.36652  1324.59954  687.4945  1664.74148  545.52671  588.94203  638.24227  332.89847  481.691  492.65099  539.8486  125.80084  167.99782  218.34481  137.7877  216.78478  132.49249  93.54857  157.1105  112.23854  136.20467  206.90948  93.45863  211.03829  244.19128  172.6548  42.94947  130.95591 |
